# Development, Validation, and Diagnostic Accuracy of the Fetal Lack of Responsiveness Scale for Diagnosis of Severe Perinatal Hypoxia

**DOI:** 10.1155/2024/9779831

**Published:** 2024-10-16

**Authors:** Luis Carlos Franco, Sandra M. Buitrago, Isabel Arbelaez, Laura F. Pinto, Daniela Blanco, María C. Pizarro, Laura Santamaria, Catalina Trillos

**Affiliations:** ^1^Facultad de medicina, Universidad de Los Andes, Bogotá, Colombia; ^2^Hospital universitario Fundación Santa Fé de Bogotá, Grupo de investigación en ginecología obstetricia y reproducción humana, Bogotá, Colombia; ^3^Sub Red Centro Oriente, Hospital Materno Infantil, Bogotá, Colombia

## Abstract

**Background:** There are limitations to predicting perinatal asphyxia, as current tools rely almost entirely on fetal cardiotocography (CTG). The fetal lack of responsiveness scale (FLORS) is a new diagnostic alternative based on the physiological phenomena associated with fetal hypoxia.

**Objectives:** The objective of this study was to develop, validate, and assess the diagnostic accuracy of the FLORS for predicting severe perinatal hypoxia (SPH).

**Study Design:** A two-phase retrospective observational cross-sectional analytical study was conducted. Phase 1 involved the formulation and retrospective validation of the FLORS. A total of 366 fetal CTG records were evaluated twice by seven readers. Phase 2 was a collaborative, retrospective, multicenter diagnostic test study that included 37 SPH and 366 non-SPH cases.

**Results:** Phase 1: A numeric, physiology-based scale was developed and refined based on expert opinions. The median time to apply the scale per reading was 38 s. Cronbach's alpha, which is a reliability measure, was significant (*p* = 0.784). The kappa index for test–retest agreement was moderate to reasonable, with a median value of 0.642. For interobserver agreement, the kappa index per variable was as follows: baseline, 0.669; accelerations, 0.658; variability, 0.467; late/variable decelerations, 0.638; slow response decelerations, 0.617; and trend to change, 0.423. Phase 2, including 37 SPH and 366 non-SPH cases, showed a sensitivity of 62.2% and specificity of 75.4% for the 2-point score, whereas the 3-point score had a sensitivity of 35.1% and specificity of 89.9%. The area under the curve (AUC) was significant at 0.73 (CI 0.645–0.818).

**Conclusions:** FLORS demonstrated significant internal consistency and observer agreement, with a promising sensitivity–specificity balance and significant AUC. Further research is needed to assess its impact on perinatal hypoxia and cesarean delivery.

## 1. Introduction

Acute peripartum or intrapartum event (APE) result from changes in gas exchange and are well-known causes of morbidity and mortality [1–5]. The incidence of APE is 5–8 per 1000 live births in low-middle income countries and 1–2 per 1000 live births in high-income settings [6, 7]. Neonatal encephalopathy and multiple organ failure are the most frequent complications. APE results in disability (4.3%) and death (12.5%) [7]. The diagnosis of APE is based on clinical criteria and umbilical cord blood analysis within the first hour after birth. These criteria include an Apgar score of ≤ 5 at 5 and 10 min, metabolic acidosis (pH ≤ 7.0) or a base deficit of ≥ 12 mMol/L, brain injury detected on MRI, or multisystem organ failure consistent with hypoxic-ischemic encephalopathy [2].

Despite numerous advances in the field, early diagnosis and timely management are still challenging tasks since the frequency of APE has not decreased [8–10]. Cardiotocography (CTG) is the standard noninvasive method for intrapartum fetal surveillance [11–14]. Multiple attempts have been made to standardize CTG interpretation through various classifications, including the American College of Obstetrics and Gynecology [15], the International Federation of Obstetrics and Gynecology (FIGO, 2015) [16], the National Institute of Healthcare and Excellence (NICE, 2017) [17], and the physiological CTG interpretation [18]. Although these classifications had a significant global impact [19], sensitivities of 32%–78% area under the curve 0.60–0.66 (estimated positive likelihood ratio: 1.47–3.51) have been reported [19, 20] for the prediction of APE. Multiple studies have made efforts to improve the diagnostic accuracy of CTG [19, 21–33]. With the introduction of new classifications, the sensitivity for APE prediction has been modestly improved to a maximum of 78%. Unfortunately, as the sensitivity increases, the specificity decreases to a maximum of 47% [20]. Alternative diagnostic methods have also been proposed, for example, fetal stimulation, fetal electrocardiogram, fetal reserve index, CTG aided by smart computer systems, cumulative deceleration area, and fetal scalp pH testing [34–36]. Due to their invasive nature, high complexity, costs, or lack of evidence, many alternative methods have not been adopted in clinical practice. Therefore, there is not an ideal choice for APE prediction since the diagnostic accuracy has not improved significantly and the cesarean rate might be affected negatively [26, 37, 38].

In order to contribute with alternative solutions to this problem, a novel approach has been formulated in the fetal lack of responsiveness scale (FLORS) (Table 1). This scale is based on recognizing the CTG physiological signs of fetal responsiveness capacity and rating the progressive loss of each of these signs rather than a systematic search for multiple pathological CTG morphologies (the classical conceptual approach to the interpretation of CTG). The FLORS is a numerical instrument that scores higher as fetal responsiveness capacity deteriorates. However, the clinical application of these new diagnostic instruments requires proper validation in terms of content, reliability, replicability, and diagnostic accuracy for outcome prediction. Therefore, this study was aimed at developing, validating, and calculating the diagnostic accuracy of the FLORS.

## 2. Materials and Methods

**Figure 1:**
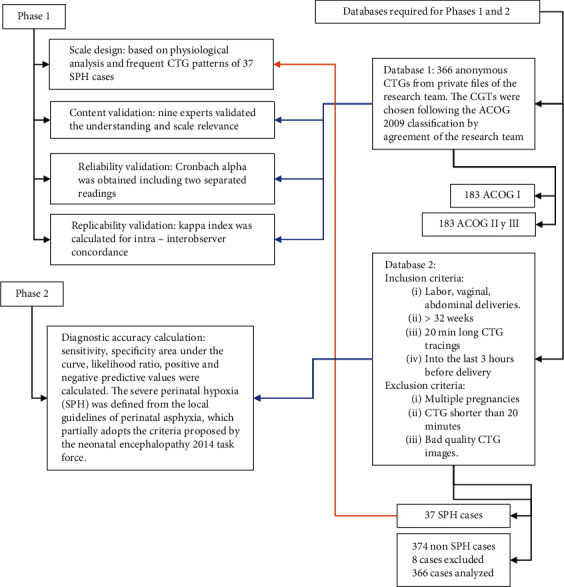
Study design algorithm.

A two-phase retrospective observational, cross-sectional analytical study was conducted in Bogotá (Colombia) using independent methods, sample sizes, and populations (Figure 1): Phase 1: development and validation of the FLORS, and Phase 2: diagnostic accuracy.

The outcome was defined postnatally as severe perinatal hypoxia (SPH) according to the Colombian consensus, the national guidelines for the diagnosis and treatment of perinatal asphyxia [3]: 5 min Apgar < 5, evidence of acidosis (pH < 7 or base excess > 12), and the registered diagnosis of perinatal asphyxia (clinical criterion of the neonatologist).

Two databases were collected retrospectively (Figure 1): the first database was a file with 366 anonymous CTGs from personal files of researchers comprising 183 CTGs classified as normal (ACOG I) and 183 classified as abnormal (ACOG II and III) according to the agreement of the research team. The first database was used for validation.

The second database contained clinical information and CTGs of 37 SPH cases and 374 non-SPH cases over 2 years from two hospitals in Bogotá (Colombia). Eight cases were excluded due to lack of CTG image (one case) or poor image quality (seven cases). The second database was used for the design of the scale (37 SPH cases) and the diagnostic accuracy phase. Vaginal and abdominal deliveries over 32 weeks in documented labor were included. Based on previous analysis [39], significant alterations in CTG readings, suggesting severe hypoxia, can occur as early as 3 h before birth. Therefore, patients whose CTG recordings fell within this timeframe were included in the study. Multiple pregnancies, CTG tracings shorter than 20 min, and poor-quality CTG images were excluded.

### 2.1. Phase 1: Development and Validation

In response to the need for a unified nomenclature of CTG readings, regardless of the presence or absence of uterine contractions, the FLORS was designed to work in both labor and nonlabor situations.

Classical elements of CTG included variability decreased (2–5 bpm) or absent (0–1 bpm), abnormal baseline (< 110 or > 160), late/variable decelerations, and sinusoidal pattern. The additional, novel variables included in FLORS were designed based on the physiological analysis of fetal hypoxia, interpretation of available evidence, and frequent CTG patterns found in the 37 SPH cases in the second database [40]. The additional variables are as follows:

*Absence of accelerations pattern*: Not included in commonly used classifications, it was found in 59% of the SPH cases. Even being a frequent finding in acidotic fetuses is also a very common physiologic pattern; therefore, in order to differentiate acidotic from fetuses in a state of quietude, the FLORS application suggests performing vibroacoustic stimulation in all cases of CTG lacking accelerations.

**Figure 2:**
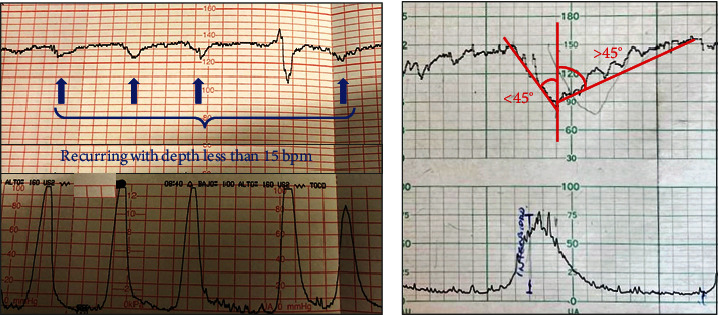
New morphologies included in FLORS. (a) Shallow decelerations morphology. (b) Example of descent angle < 45° and recovery angle > 45°.

*Slow-response morphology pattern*: This was found in 44% of SPH cases. It contributes to the understanding of decelerations as a sign of fetal responsiveness to the pressure exerted on the head within the pelvis [41] and other mechanisms that are yet to be established. In any case, we believe that deceleration indicates autonomic responsiveness. The presence of late and variable decelerations (lack of coincidence with contraction) indicates CTG alerts [20]. In addition, FLORS gives equal importance to the morphology of the decelerations, regardless of its coincidence with the uterine contraction. Consequently, the variable “slow-response morphology decelerations” is introduced. A fetus with a tendency to deteriorate will progressively lose its ability to decelerate, initially widening the deceleration morphology (evaluated by the finding or wide fall/recovery angles > 45°) (Figure 2), followed by shallow decelerations (a pattern of decelerations but less than 15 bpm depth) (Figure 2); ultimately, the fetus would lose its deceleration capacity, showing a flat trace morphology.

*Trend-to-change speed pattern*: It recognizes that the hypoxia process in the fetus can be acute, subacute, or chronic [18]. The FLORS rates the acute loss of responsiveness signs along a 20 min tracing. To standardize the evaluation of this variable, FLORS proposes to interpret the CTG by rating the two halves independently (10 min each). A higher second-half rate indicates an accelerated trend of deterioration. This pattern was observed in 31% of SPH cases.

*Validation process*: The content validation of the scale was carried out by nine experts from three different hospitals. An expert was defined as a certified specialist in obstetrics and gynecology with more than 5 years of experience in the clinical evaluation of CTG. The understanding of the questionnaire and its relevance in the clinical context were assessed, and based on every expert opinion, the questions were adjusted, explanatory graphs were added, and scale domains were reorganized.

A CTG reading pilot study was conducted with the same nine experts. An information bias was detected because obstetricians, due to their experience, tended to adjust the reading to their first impression of the CTG. Consequently, to guarantee that any healthcare provider would be able to apply, the scale introduced the definition of a “reader,” as a last-year medical student who had passed basic obstetrics and epidemiology courses. The criteria used were “a person with basic but sufficient knowledge in obstetrics.” The requirement of having passed a basic epidemiology course was introduced to ensure that the reader understands the importance of the methodological process for data reliability.

For the first phase sample size calculation, a pilot test was carried out by evaluating 20 CTGs twice by 10 readers (400 readings). The time between the two reading sessions was 48 h. Cronbach's alpha obtained was 0.56. The sample size was calculated to reach Cronbach′s alpha ≥ 0.7, a power of 80%, and a confidence level = 95%. The result was 338 CTGs. To reduce the sampling error, a final sample size of 366 CTGs was decided (first database). For statistical analysis, software R v7.9 was used.

*Data acquisition*: Seven volunteer readers were recruited for the scale application. They received 30-min standardized instruction on fetal CTG nomenclature and application of the FLORS, including group readings of random CTGs to unify concepts. The time required to read and load interpretations in a digital form was measured. Each of the 366 CTGs was read twice (5124 readings) in a 48-h interval between the first and second reading sessions. The interpretation of each CTG was reported in a digital format, and a double entry was required for typing error control.

IBM SPSS V 25 licensed to the Universidad de Los Andes was used for statistical analysis. For reliability validation, Cronbach's alpha was calculated per reader, and mean values were obtained, including both the first and second readings. For intraobserver concordance, kappa indices were calculated by comparing the first and second readings for every variable of the scale. For interobserver agreement, kappa indices were calculated for every variable, and for every single comparison between readers, median values and ranges were reported. For the reliability measure of the kappa index results, Fisher's exact test was obtained.

### 2.2. Phase 2: Diagnostic Accuracy Calculation

A collaborative, blinded, multicenter study was conducted to establish the diagnostic precision of the FLORS. The protocol was approved by the ethics committees of two institutions, Fundacion Santa Fe de Bogotá (CCEI-14622-2021) and Subred Centro Oriente (CEI. 067/2022). Institutional delivery databases were consulted, and clinical data and CTG images were obtained. The RedCap database (licensed for Universidad de los Andes) was used to upload and store the data. The sample sizes were 360 non-SPH and 37 SPH cases. Sample estimators: incidence, 5/1000 live births; power, 80%; and error, 5%. Two volunteers were recruited as “readers.” Readers received 30-minute instruction on the application of FLORS, which included group reading of 15 CTGs (not included in the sample) to unify concepts. The sample was randomized, and the outcomes were blinded. The readers applied the FLORS and provided each CTG final interpretation by consensus. The readers loaded the information obtained in digital form twice for error control.

**Figure 3:**
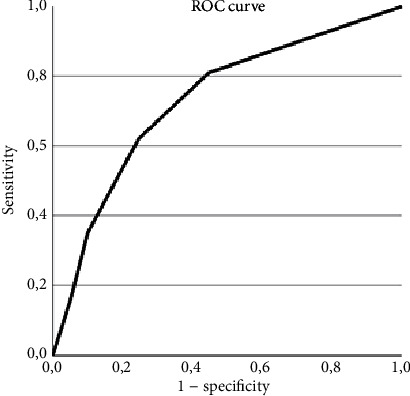
FLORS ROC curve.

Microsoft Excel (V16.71 licensed for Universidad de los Andes) was used for sensitivity, specificity, predictive values, and likelihood ratio calculations (Table 2). The receiver operating characteristic (ROC) curve (Figure 3) and the area under the curve were calculated. The most discriminative cutoff point of the scale was found by the value with the shortest distance to the 0,1 point on the ROC curve $d=\sqrt[{2}]{{\left(1-sensitivity\right)}^{2}+{\left(1-specificity\right)}^{2}}$. IBM SPSS (V28 licensed for Universidad de los Andes) was used for the statistical analysis.

## 3. Results

A total of 5124 readings were obtained during the validation phase. The median scale application time per reading was 38 s (range: 00:12–02:10), including the time required for digital form typing. After the first 20 readings, a tendency to shorten the reading times was observed (learning curve).

The median Cronbach's alpha was 0.777. These results indicate that the scale was reliable for all readers. Concordance between the first and second readings was found, and the kappa index was significant for every variable (Table 3), with reasonable to moderate concordance. Agreement between the readers was calculated. The kappa index showed significant concordance (reasonable to moderate) when comparing each reader's results (Table 3). Fisher's exact test was significant (*p* < 0.05) for each comparison.

For the analysis of diagnostic accuracy, 37 SPH and 374 non-SPH records were included (Table 4), 8 non-SPH were excluded, and 37 SPH and 366 non-SPH were analyzed. The ROC curve was obtained (Figure 3), and the area under the curve was calculated (0.73) (CI 95% =0.645–0.818). The scale cut point of two (≥ 2) had the most discriminative value, with the best relationship between sensitivity and specificity (Figure 3 and Table 2; Supporting Information (available here)).

## 4. Discussion

The scale was reliable since Cronbach's alpha was > 0.7 for every variable. No other reported reliability of CTG interpretation was found in the literature review. The kappa indexes obtained showed significant intra- and interobserver concordance; however, even with very promising results, they cannot be compared with the concordance degree reported by other authors [20] because the statistical methods used were different and a fair comparison demands analysis of the same sample, readers, and methodology.

Although a fair discriminatory capacity comparison with other classifications requires unifying the research methods, FLORS seems to have slightly higher diagnostic accuracy than commonly used classifications, with the versatility of a numeric scale [20].

The AUC and likelihood ratio should be carefully discussed in terms of the relationship between sensitivity and specificity. The FLORS AUC showed a statistically significant value (0.73) for the first time as an SPH prediction tool. The main strength of FLORS is its quite high AUC value, indicating a reasonable discriminant ability. Using the optimum cut-point of ≥ 2, we correctly identified 74% of individuals in this study. This result is higher than reported for other classifications and should motivate the design of novel clinical strategies for fetal surveillance based on the tendency in time of the FLORS. Regarding the FLORS likelihood ratios (2.53–3.48), even slightly higher than those estimated for other classifications, it might be interpreted as not significant. We believe that the objective of FLORS, or any diagnostic tool, should not be to demonstrate a higher posttest probability; it would be too late for the newborn since SPH is a catastrophic outcome. Efforts in clinical practice should aim to diagnose the process of hypoxia rather than asphyxia in a timely manner.

The cutoff definition results were consistent with the original interpretation of the FLORS. A scale score of 2 points indicates a significant but moderate risk of SPH, uterine resuscitation measures should be performed, and a second CTG should be evaluated (41–43); a scale score of 3 points also shows significant discriminative values, with a higher risk of SPH, which indicates interruption or shortening of labor.

The FLORS is a promising tool for the interpretation of CTG. Its diagnostic accuracy indicates that its application may potentially reduce the incidence of SPH. The FLORS was validated for readers with basic sufficient training, so we believe that if the scale is applied by more trained readers, the instrument should maintain or even improve its performance. Further studies are required to compare its diagnostic accuracy with that of other classifications. Prospective research is needed to evaluate its impact on SPH incidence and cesarean section rates.

Regarding the limitations of the present study, FLORS was applied retrospectively in a population that had already been assessed by ACOG classification, and clinical decisions were made (e.g., in utero resuscitation and interruption of labor); therefore, FLORS real accuracy cannot be fully assessed, and prospective studies are required. The FLORS was validated in Spanish, which might be considered a limitation for its application in different language settings.

The implementation of FLORS is expected to be better suited in low-resource clinical settings, but the conceptual contributions of this study might be used to improve clinical practice worldwide and to feed the algorithms of artificial intelligence where they are operating.

## 5. Conclusion

The FLORS is an easy-to-use, fast-to-learn, reliable tool with adequate intra- and interobserver agreement that shows a promising capacity to predict SPH. Thus, it is an alternative for use in clinical settings. Further prospective studies are required to evaluate its impact on outcomes of public health interest.

## Figures and Tables

**Table 1:** Fetal lack of responsiveness scale.

**Variable**	**Score**
First domain: Loss of responsiveness to hypoxia	
Fetal tachycardia or bradycardia	1 = baseline > 160 or < 110 bpm
Variability	1 = 2–5 bpm
	2 = 0–2 bpm or sinusoidal pattern
Absence of accelerations	1 = < 2/20 min
Contraction/deceleration mismatch	1 = late or variable recurrent^a^ decelerations
Slow response morphology decelerations	1 = > 45° drop angle or recovery angle, prolonged decelerations (2–10 min) or shallow decelerations morphology (recurrent^a^ with less than 15 bpm depth)

Second domain: Trend-to-change speed	
The reading of the tracing must be divided into two halves, comparing the tracing scores of the first 10 min with the final 10 min	1 = the score for the second half increases by 1 point
	2 = the score for the second half increases by 2 or more points

Third domain: Response to in utero resuscitation (IUR)	
In all cases of suspected alteration of fetal well-being, in utero resuscitation measures (IUR) will be applied^b^	1 = loss of response capacity criteria persists or worsens

Interpretation	
1 = signs of well-being, expectant management, routine surveillance 2 = possible alteration of well-being—In utero resuscitation maneuvers and reassess CTG^b^ 3 = significant changes require urgent delivery or cesarean section 4 = major changes require emergency delivery or cesarean section	

^a^Recurrent is defined as present with at least 50% contractions.

^b^Hydration, side position, oxygen supplementation, interruption of uterotonic medications, and consider tocolysis.

**Table 2:** Diagnostic accuracy values of the most discriminative scale scores.

**Scale score cut point**	**Sensitivity**	**Specificity**	**Positive predictive value**	**Negative predictive value**	**Positive likelihood ratio**	**Negative likelihood ratio**
≥ 1	81%	55.1%	15.46%	96.65%	1.81	0.34
≥ 2	62.2%	75.4%	20.35%	95.17%	2.53	0.50
≥ 3	35.1%	89.9%	26.00%	93.20%	3.48	0.72
≥ 4	16.21%	94.8%	24%	91.8%	3.12	0.88

**Table 3:** Concordance results per variable.

**Variable**	**Intraobserver**		**Interobserver**	
	**Kappa index**	**Concordance level (McHugh, 2012)**	**Kappa index**	**Concordance level (McHugh, 2012)**
Baseline	0.767	Moderate	0.6695	Moderate
Accelerations	0.715	Moderate	0.6555	Moderate
Variability	0.513	Reasonable	0.4755	Reasonable
Late/variable decelerations	0.706	Moderate	0.638	Moderate
Slow response decelerations	0.652	Moderate	0.617	Moderate
The trend to change speed	0.549	Reasonable	0.423	Reasonable

**Table 4:** Phase 2 demographic information.

**Outcome**	**Non-SPH cases (** **n** = 374**)**	**Perinatal SPH cases (** **n** = 37**)**	**All cases**
Maternal age			
Mean (standard deviation)	28.13 (7.02)	28.97 (7.77)	28.2 (7.07)
Socioeconomic level—Poorest to richest			
1	76 (20.32%)	24 (24%)	100 (24.33%)
2	98 (26.2%)	7 (18.92%)	105 (25.55%)
3	72 (19.25%)	1 (2.7%)	73 (17.76%)
4	33 (8.82%)	1 (2.7%)	34 (8.27%)
5	62 (16.58%)	1 (2.7%)	63 (15.33%)
6	33 (8.82%)	3 (8.11%)	36 (8.76%)
Education level			
No formal education	4 (1.07%)	1 (2.7%)	5 (1.22%)
Middle school	51 (13.64%)	6 (16.22%)	57 (13.87%)
High school	133 (35.56%)	21 (56.76%)	154 (37.47%)
Technical	35 (9.36%)	3 (8.11%)	38 (9.25%)
Professional	94 (25.13%)	5 (13.51%)	99 (24.09%)
Postgraduate	47 (12.57%)	0 (0%)	47 (11.44%)
Gestational age (weeks)			
Mean (standard deviation)	38.27 (1.45)	38.54 (1.87)	38.3 (1.49)
Mode of delivery			
Vaginal	262 (70.05%)	22 (59.46%)	284 (69.1%)
Cesarean section	102 (27.27%)	11 (29.73%)	113 (27.49%)
Instrumented birth	10 (2.67%)	4 (10.81%)	14 (3.41%)
SPH-related comorbidities			
None	311 (83.16%)	27 (72.97%)	338 (82.24%)
Intrauterine growth restriction	23 (6.15%)	2 (5.41%)	25 (6.08%)
Intra-amniotic infection	1 (0.27%)	3 (8.11%)	4 (0.97%)
Preterm premature rupture of membranes	7 (1.87%)	1 (2.70%)	8 (1.94%)
*Abruptio placentae*	1 (0.27%)	0 (0.00%)	1 (0.24%)
Umbilical cord prolapse	2 (0.53%)	1 2.70%	3 (0.73%)
Other umbilical cord pathologies	0 (0.00%)	1 (2.70%)	1 (0.24%)
Preeclampsia	12 (3.21%)	2 (5.41%)	14 (3.41%)
Third trimester bleeding	0 (0.00%)	1 (2.70%)	1 (3.41%)
Oligohydramnios	4 (1.07%)	0 (0.00%)	4 (0.97%)
*Time last CTG until birth*			
Mean in minutes (standard deviation)	86.31 (48.01)	106.72 (57.16)	85.42 (49.28)

## Data Availability

The data that support the findings of this study are available on request from the corresponding author. The data are not publicly available due to privacy or ethical restrictions.

## Supporting Information

Supporting Information Additional supporting information can be found online in the Supporting Information section. Excel table with the results of the second phase and the calculation of the results for diagnostic accuracy.
